# Massive gene loss in mistletoe (*Viscum*, Viscaceae) mitochondria

**DOI:** 10.1038/srep17588

**Published:** 2015-12-02

**Authors:** G. Petersen, A. Cuenca, I. M. Møller, O. Seberg

**Affiliations:** 1 Natural History Museum of Denmark, University of Copenhagen, Sølvgade 83, DK-1307 Copenhagen K, Denmark; 2 Department of Molecular Biology and Genetics, Aarhus University, Forsøgsvej 1, DK-4200 Slagelse, Denmark

## Abstract

Parasitism is a successful survival strategy across all kingdoms and has evolved repeatedly in angiosperms. Parasitic plants obtain nutrients from other plants and some are agricultural pests. Obligate parasites, which cannot complete their lifecycle without a host, may lack functional photosystems (holoparasites), or have retained photosynthesis (hemiparasites). Plastid genomes are often reduced in parasites, but complete mitochondrial genomes have not been sequenced and their mitochondrial respiratory capacities are largely unknown. The hemiparasitic European mistletoe (*Viscum album*), known from folklore and postulated therapeutic properties, is a pest in plantations and forestry. We compare the mitochondrial genomes of three *Viscum* species based on the complete mitochondrial genome of *V. album*, the first from a parasitic plant. We show that mitochondrial genes encoding proteins of all respiratory complexes are lacking or pseudogenized raising several questions relevant to all parasitic plants: Are any mitochondrial gene functions essential? Do any genes need to be located in the mitochondrial genome or can they all be transferred to the nucleus? Can parasitic plants survive without oxidative phosphorylation by using alternative respiratory pathways? More generally, our study is a step towards understanding how host- and self-perception, host integration and nucleic acid transfer has modified ancestral mitochondrial genomes.

Mitochondria, originally captured α-proteobacteria, produce energy through respiration and are thought to be required for eukaryote complexity and the evolution of multi-cellularity. Mitochondria house key metabolic pathways (e.g., the tricarboxylic acid cycle (TCA), Fe-S-protein biogenesis, co-enzyme production) and mediate forms of programmed cell-death, anti-microbial defense and stress responses. Mitochondrial genomes vary remarkably in size and structural heterogeneity, particularly in protists, lower metazoans and plants, and there are even reports of eukaryotes in which the mitochondrial genome is almost certainly absent^1^. While the animal mitochondrial genome size of 15–18 kb is almost constant, that of seed plants varies from 208 kb to more than 11 mb^2^,^3^. A significant part of the increase in size in plants is due to inter-organellar transfer^4^,^5^,^6^ and, at least in some cases, exogenous DNA^2^,^7^. However, imported genes usually degrade into pseudogenes^8^. For these reasons ~5150 complete animal mitochondrial genomes have been sequenced and assembled, but only 58 from seed plants. The differences in size are not reflected in numbers of protein-coding genes. For example, the *Arabidopsis* mitochondrial genome is 22 times larger than that of humans but only encodes 2.5 times as many proteins (13 and 31, respectively)^9^, and even the smallest and largest genomes of nonparasitic seed plants contain almost the same number of genes. The smallest number of protein-coding mitochondrial genes hitherto reported in a seed plant is 25^10^. Though complete mitochondrial genomes from holoparasitic species of Rafflesiaceae have not been assembled, sequencing data do not suggest gene loss^11^,^12^.

The hemiparasitic genus mistletoe (*Viscum*) consists of ~100 species parasitic on a large variety of hosts (Fig. 1). While it is known that the chloroplast genomes of parasites may be reduced together with the loss of photosynthetic activity^13^, we show here that the number of proteins encoded by the mitochondrial genomes of three *Viscum* species is significantly smaller than seen in any other seed plant (Fig. 2). This indicates that the chloroplast and mitochondrial genomes of parasitic plants share parallel fates and raises questions about the functions of their mitochondria.

Roughly half the proteins encoded in the mitochondria of seed plants are involved in the respiratory chain and tricarboxylic (TCA) acid cycle, and are therefore usually considered essential^5^. Proteins of the respiratory chain belong to five enzymatic complexes. Complexes I, III and IV function in proton translocation and are probably tightly associated in a supramolecular assembly, whereas complex II conveys electrons to the respiratory chain and complex V uses the proton gradient to phosphorylate ADP to ATP^14^.

Complex I, NADH-ubiquinone oxidoreductase, is the largest complex in the respiratory chain. It is responsible for the first steps in electron transport which lead to the translocation of four protons between the matrix and the intermembrane space, though further enzymatic activities have also been postulated^15^. In *Arabidopsis*, complex I is composed of 48 subunits^16^. Typically, nine of them are encoded in the mitochondrial genome^17^ (*nad1, 2, 3, 4, 4L, 5, 6, 7, 9*) and are invariably present in nonparasitic seed plants^4^ (Fig. 2). However, none of these genes are present in the mitochondrial genome of the three *Viscum* species although a few smaller, degenerated fragments may be identified (Extended Data Table 1).

Complex II, succinate dehydrogenase (SDH), is the only enzyme with a role in both the TCA cycle and electron transport. In *Arabidopsis* it is composed of eight subunits, four of them plant specific. Two of the subunits are encoded in the mitochondrial genome, and the function of four of the remaining subunits remains unknown^18^. Whereas the amino acid sequences of SDH1 and SDH2 are fairly well conserved across kingdoms, those of the membrane anchor proteins SDH3 and SDH4 are very diverse. Only *sdh3* and *sdh4* are found in mitochondrial genomes of seed plants, though they are often missing or pseudogenized (Fig. 2). A short fragment of *sdh3* is found in all three *Viscum* species, but only *V. album* includes a fragment of *sdh4*.

Complex III, the cytochrome *bc*_*1*_ complex, consists of 10–11 subunits^19^ of which only one is encoded in the mitochondrial genome^17^. It oxidizes the ubiquinol produced by complex I and II by transferring electrons from ubiquinol to cytochrome c and pumping protons across the membrane. Apocytochrome *b* (*cob*) is without exception found in all seed plant mitochondrial genomes and is present in the three *Viscum* species (Fig. 2), but highly divergent from other seed plant *cob* sequences (Extended Data Fig. 1).

Complex IV, cytochrome c oxidase, is encoded by 11 genes, three encoded in the mitochondria^17^. It catalyzes the complete reduction of oxygen to water, and promotes proton translocation across membranes to help form the electrochemical gradient. The *cox1*, *cox2* and *cox3* genes are found in all seed plants except in certain legumes^20^ in which a functional *cox2* copy is transferred to the nuclear genome^21^ (Fig. 2). The three *Viscum* species all have the three *cox*-genes, but like *cob* these genes are highly divergent (Extended Data Fig. 2). Whereas *cox2* in other seed plants include zero to two *cis*-spliced introns, the first intron in *V. album* is *trans*-spliced and both introns appear *trans*-spliced in *V. crassulae* and *V. minimum*.

Complex V, ATP or F_1_F_0_-ATP synthase, is encoded by 16 genes, five of which are mitochondrial in *Arabidopsis*^17^ and all other seed plants (Fig. 2) with one possible exception^4^. It uses the proton gradient produced by complexes I, III and IV to synthesize ATP from ADP^22^. Two of the five genes of complex V encoded in the mitochondria of all other known seed plants are missing in the three *Viscum* species and the remaining genes are highly divergent (Extended Data Fig. 3).

Apart from the respiratory chain genes, seed plant mitochondrial genomes all include four genes related to cytochrome biogenesis (*ccmB*, *C*, *Fc* and *Fn*) and a maturase-related protein gene (*matR*). Whereas all three *Viscum* species have retained *matR*, they have lost one or two of the cytochrome biogenesis genes (Fig. 2). Most seed plant mitochondria also include a membrane transport protein gene (*mttB*). This is pseudogenized in the *Viscum* species, is missing in other seed plants (Fig. 2, Extended Data Table 1), and appears to be non-essential. In addition, most seed plants include a variable number of ribosomal protein genes (up to 15), but none of them are ubiquitous^5^. We found a single gene in *V. album* (*rps12*), but the absence of all genes in the other two species of *Viscum* is remarkable. So far, ribosomal RNA genes have been found in the mitochondrial genomes of all seed plants (Fig. 2), and cotranscription of the 18S-5S rRNA cluster has been considered indispensable^23^. While seed plant mitochondrial genomes never have all 20 tRNAs necessary for protein synthesis, the presence of only 3–4 tRNA genes in the *Viscum* mitochondrial genomes is highly unusual^3^ (Extended Data Table 1).

However, we were unable to locate 5S rRNA in any of the three *Viscum* species, and the recovered 26S rRNA-like sequences are so divergent that the genes are unlikely to be functional. The identified sequences of the 18S rRNA genes are also very different from those of all other seed plants suggesting that they too are not functional. Additionally a number of ribosomal proteins are usually encoded in the plant mitochondrial DNA, but in *Viscum album* only one complete ribosomal gene and one fragment is found, and none in the other two *Viscum* species. While seed plant mitochondrial genomes never have all 20 tRNAs necessary for protein synthesis, the presence of only 3–4 tRNA genes in the *Viscum* mitochondrial genomes is highly unusual^3^ (Extended Data Table 1). Thus, the three *Viscum* mitochondrial genomes are not only exceptionally gene poor, but those present are almost all extremely divergent from those of other seed plants (Fig. 3, Extended Data Figs 1–3). Nonetheless, a phylogenetic analysis of 10 of the protein-coding genes places the three *Viscum* species in their traditional position sister to Caryophyllales and the asterids, but on an exceptionally long branch reflecting the highly divergent sequences of the genes (Fig. 3). Individual gene trees (Extended Data Figs 1–4) reveal that only two genes (*matR* and *ccmB*), not part of the respiratory chain, are not unexpectedly divergent. This may indicate that they are the only truly functional genes in their mitochondrial genomes. In general, it appears that only respiratory complexes III and IV are fully encoded in the mitochondrial genomes of the three *Viscum* species, but their potentially pseudogenized state and the paucity of the remaining respiratory genes is a strong indication that none of them are functional. And even if they are functional, *Viscum* mitochondria would be unable to perform protein biosynthesis unless all the missing rRNA, ribosomal proteins and tRNAs are imported.

If legumes have successfully transferred the *cox2* gene to the nucleus^20^,^21^, is it then possible that *Viscum* has transferred a whole suite of genes and retained a fully functional electron transport chain and oxidative phosphorylation? This would explain how the seedlings survive prior to establishing physical contact with a host, which would otherwise be very difficult, but our sequencing depth was insufficient to decisively detect nuclear transfers. These questions will be pursued in transcriptome and proteome studies possibly coupled with deeper sequencing of *Viscum* nuclear genomes, though the latter may be impeded by their enormous size^24^.

The alternative to the transfer of most or all the mitochondrial genes to the nucleus is that *Viscum* mitochondria have experienced a reductive evolution similar to that observed for hydrogenosomes and mitosomes in anaerobic ciliates and fungi^25^. The mitosomes appear to lack the ATP-generating pathways and have a simpler structure as they lack cristae. Nonetheless they have been retained as independent organelles probably to provide the cell with important metabolic intermediates, iron-sulfur clusters, coenzymes, etc. In this scenario, *Viscum* cells would obtain their ATP from photosynthesis (which starts immediately following germination in *Viscum album* seeds^26^) and eventually from glycolytic degradation of sugars imported from the host after the establishment of the haustorium. In the aerobic *Viscum* cells, cytosolic NADH and ATP could then be used to fuel respiratory metabolism, for instance by energizing the inner mitochondrial membrane, which is necessary to ensure a controlled exchange of metabolites between the matrix and the cytosol.

To speculate further, cytosolic NADH (or NADPH) could potentially be oxidized by one of the alternative NAD(P)H dehydrogenases located on the outer surface of the inner mitochondrial membrane and the electrons via ubiquinone go to the alternative oxidase, which reduces oxygen on the inner surface. All of the alternative NAD(P)H dehydrogenases as well as the alternative oxidase are nuclear-encoded so they would be unaffected by the loss of virtually the full mitochondrial genome. There is co-expression evidence to suggest that each external NAD(P)H dehydrogenase in plant mitochondria is associated with a particular alternative oxidase isoform, although evidence for a specific functional interaction is still lacking^27^. The net result would be a transfer of two electrons per NAD(P)H molecule from the outside to the inside of the mitochondrion, which would create a membrane potential. At the same time a proton will be released on the outer surface and two protons consumed on the inner surface setting up a proton gradient with a more basic matrix. Thus, an electrochemical proton gradient across the inner mitochondrial membrane is formed similar to that formed by the complete electron transport chain. This alternative mechanism of inner membrane energization would be less efficient because only two protons are transferred per two electrons compared to six protons when the electrons from external NADH pass through complexes III and IV^27^, but it would be able to energize metabolite exchange across the inner membrane (e.g., import of ATP). In contrast, it would not be coupled to ATP synthesis because of the absence of the ATP synthase (Complex V). Similarly, but without energization of the inner membrane, NADH produced in the matrix by the TCA cycle could be reoxidized by an internal alternative NADH dehydrogenase linked to another alternative oxidase isoform, thus allowing the continued function of the TCA cycle. Note that in the scenario we are considering here, the five respiratory complexes are missing including the TCA cycle enzyme succinate dehydrogenase (Complex II). The TCA cycle would therefore have to use alternative flux modes^28^ to produce all the important metabolic intermediates.

Interestingly, parallel reductions in the plastid gene complements of parasitic plants have been observed, most drastically in Rafflesiaceae where the entire plastome may be lost^12^, pointing at a correlation between diminished photosynthetic capacity and different degrees of nutritional dependence upon the host^13^. Surprisingly, our data from the plastid genome of *Viscum* e.g, showing loss of all NADH dehydrogenase genes, are in line with this trend^29^.

In general our study raises a series of challenges faced by any parasitic plant related to recognizing self and non-self, host integration, alternative respiratory pathways and the extent of ancestral nucleic acid transfer in the seed plant tree of life.

## Methods Summary

DNA from intact mitochondria isolated from *Viscum album* leaves was extracted using a CTAB procedure and sequenced using 454 GS FLX Titanium technology. The complete mitochondrial genome was assembled using Newbler and bb.454contignet software, and coding regions identified by BLAST searches against GenBank and local data bases. From two other *Viscum* species, *V. crassulae* and *V. minimum*, total genomic DNA was extracted and sequenced using Illumina/HiSeq technology. Sequence reads were used to extract mitochondrial gene sequences only. Sequences from 10 mitochondrial genes identified in all or just one species of *Viscum* were included in a phylogenetic analysis together with sequences from other seed plants from which whole mitochondrial genome sequences are available. Individual gene trees were also constructed.

## Methods

### Plant material

Fresh material of three species of *Viscum* L. was collected from specimens grown in the Botanical Garden, Natural History Museum of Denmark, University of Copenhagen, Denmark. In the garden *Viscum album* L. is a parasite on *Malus* Mill. sp. and the two endemic, South African species *V. crassulae* Eckl. & Zeyh. and *V. minimum* Harv. are parasites on *Portulacaria afra* Jacq. and *Euphorbia mammillaris* L., respectively. Voucher specimens are deposited in the herbarium (C) of the Natural History Museum of Denmark (*V. album*: C2546, *V. crassulae*: C2553, *V. minimum*: C2884).

### DNA extraction and sequencing

Green leaves were used for DNA extraction of *V. album*. Intact mitochondria were isolated by centrifugation following a modified protocol^30^, and using DNAase I to digest nuclear and other DNA contaminants. Mitochondrial DNA was extracted using CTAB and a regular chloroform-isoamyl alcohol DNA isolation protocol. Whole genome amplifications were carried out using repli-g kit (Qiagen) after the manufacturer’s protocol. A GS FLX Titanium (Roche, USA) shot gun library was constructed and sequenced at the National High-throughput DNA Sequencing Centre, U. Copenhagen in a quarter of a GS PicoTiterPlate according to the manufacturer’s instructions.

Green leaves were also used for DNA extraction of *V. crassulae*, but to minimize the risk of host tissue contamination, green seeds were used for DNA extraction of the minute *V. minimum*. In both cases ca. 100 mg of fresh tissue was used for total genomic DNA extractions using a standard CTAB method^31^. Illumina short-insert, paired-end libraries with average insert sizes of 500 bp were constructed and each sample was run in 1/16 of a lane on an Illumina HiSeq 2500.

### Sequence analysis

A total of 60,389 sequences (average size 339 nt) from *V. album* were assembled using Newbler 2.3 (454 Life Sciences Corp, CT, USA) using default settings. A total of 38 contigs >100 nt were obtained, with the largest contig of 116,034 nt. The bb.454contignet software^32^ was used to visualize the contig connections and join the contigs. Contig connections were also checked against raw reads. The total assembled genome has a size of 565,432 nt and was assembled into a linear structure. An alternative assembly would break the genome into two submolecules, one circular and the other linear. Average sequence coverage of the mitochondrial genome was 18×. The genome sequence is deposited in GenBank under accession number KJ129610.

Coding regions were identified by BLASTX searches performed against a local database including nucleotide sequences for all coding genes from 20 plant mitochondrial genomes available in GenBank. The exact gene and exon boundaries were determined by alignment of homologous genes from available annotated plant mitochondrial genes. rRNA and tRNA genes were identified by BLASTN searches against a local database including all rRNA genes and a database including all tRNA genes from 20 available land plant mitochondrial genomes. tRNAs were annotated using tRNAscan-SE^33^,^34^.

A total of 24 and 40 million sequences of length 101 nt were obtained from *V*. *crassulae* and *V. minimum*, respectively. Average coverage of mitochondrial sequences was ca. 35× for both samples. Using the complete mitochondrial genome of *V*. *album* as a reference, the sequences from *V. crassulae* and *V. minimum* were mapped in Geneious ver. 6 using the Map to Reference function with Medium-Low sensitivity and 5 times iteration. Sequences mapping to identified, complete or partial genes were extracted, reassembled in individual loci and extended again with the Map to Reference function. A data base of genes from other completely assembled mitochondrial genomes was used as reference sequences to search for genes not present in *V. album* mitochondrial loci. Identified complete and partial genes and flanking sequence are deposited in GenBank under accession numbers KJ146758-KJ146774 (*V. crassulae*) and KJ146758-KJ146774 (*V. minimum*) (see Extended Data Table 1 for further details).

### Phylogenetic analysis

Sequences from 10 protein coding genes (*atp1*, *atp6*, *atp9*, *ccmB*, *ccmC*, *cob*, *cox1*, *cox2*, *cox3*, *matR*) present in full length in at least one species of *Viscum* were used for a phylogenetic analysis including sequences from 28 other seed plants for which complete mitochondrial genomes are available in GenBank. Alignments were constructed manually using Mesquite ver. 2.75^35^ taking codon and amino acid sequence conservation into account. Due to alignment ambiguity two genes present in all or just one species of *Viscum* (*ccmFn* and *rps12*) were not included in the phylogenetic analyses. For the same reason we did not attempt to use the ribosomal RNA gene sequences. Maximum likelihood (ML) analyses of individual genes were done at the Cyperinfrastructure for Phylogenetic Analysis (CIPRES; www.phylo.org) running RAxML-HPC Blackbox ver. 8.0.0^36^. Default options were used, except that the GTR plus GAMMA + I model was applied to each gene when run individually. A combined ML analysis of all genes was performed as above except that the input file was treated both as unpartitioned and as divided into 10 partitions with very similar results. The ML trees were edited using the program FigTree ver. 1.4.0 (tree.bio.ed.ac.uk/software/figtree).

## Additional Information

**Accession codes:** The complete mitochondrial genome sequence of *V. album* is deposited at GenBank under accession number KJ129610, and sequences of genes and named gene fragments from *V. crassulae* and *V. minimum* under accession numbers KJ146758-KJ146774 and KJ146758-KJ146774, respectively. 

**How to cite this article**: Petersen, G. *et al.* Massive gene loss in mistletoe (*Viscum*, Viscaceae) mitochondria. *Sci. Rep.* **5**, 17588; doi: 10.1038/srep17588 (2015).

## Figures and Tables

**Figure 1:**
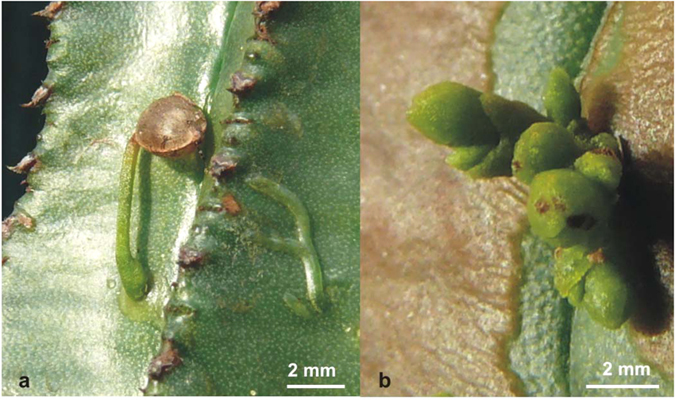
*Viscum minimum* parasitizing succulent *Euphorbia*. (**a**) The *Viscum* seedling to the left has established contact with the host through a haustorium. Endophytic growth of the parasite is visible to the right just beneath the host, *Euphorbia horrida*, epidermis; (**b**) Mature, green shoots of *Viscum minimum* on *Euphorbia mammillaris*. (Photo: Gitte Petersen).

**Figure 2:**
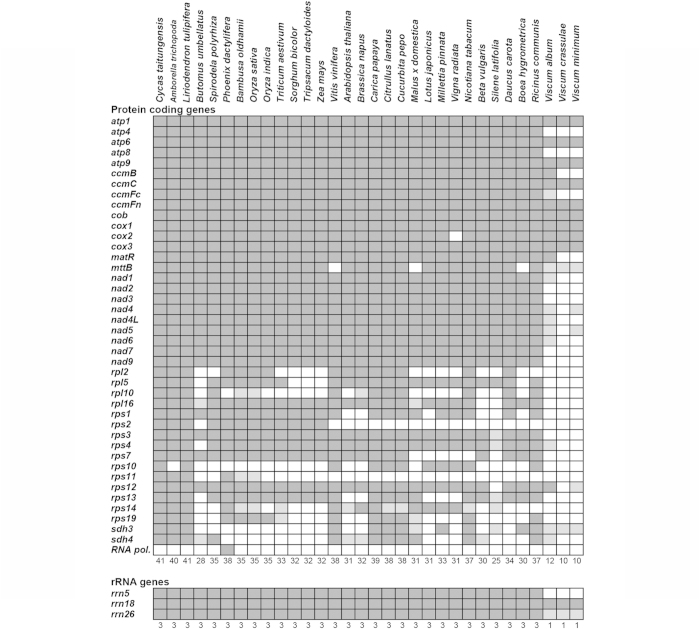
Overview of gene content in the mitochondrial genome of *Viscum* and 29 other seed plants. Dark grey fields indicate presence of the gene. Light grey fields indicate pseudogenes or gene fragments. The rrn18 genes are scored as functional with doubt. See Extended Data Table 1 for details of the genes (including tRNAs) found in *Viscum*.

**Figure 3:**
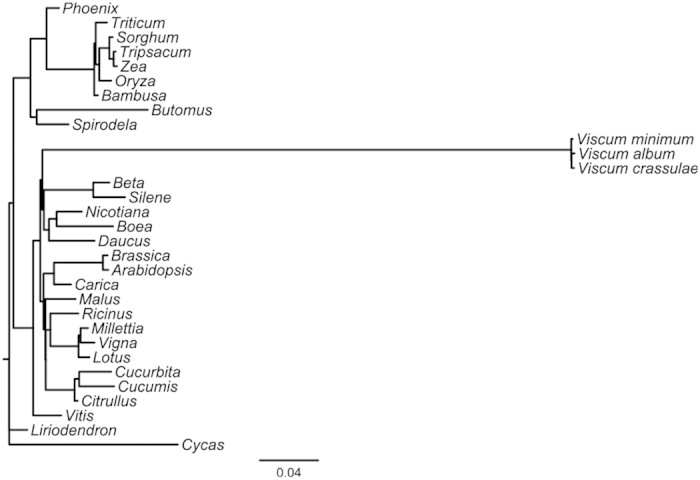
Phylogenetic tree based on sequence data from 10 mitochondrial genes. The position of *Viscum* is in agreement with models of angiosperm phylogeny, but the long branch indicates that the *Viscum* gene sequences are extremely divergent compared to other plants. See Extended Data Figs 1–4 for individual gene trees. Scale bar, substitutions per site.
